# A Single Liver Metastasis From Adenoid Cystic Carcinoma of the Parotid Gland: Case Report

**DOI:** 10.1177/2324709619879631

**Published:** 2019-09-26

**Authors:** Ines Zemni, Nesrine Tounsi, Imene Bouraoui, Maher Slimene, Ghada Sahraoui, Mohamed Ali Ayadi, Riadh Chargui, Khaled Rahal

**Affiliations:** 1 Department of surgical oncology, Salah Azaiz Institute, University Tunis El Manar, Tunis, Tunisia; 2 Department of pathology, Salah Azaiz Institute, University Tunis EL Manar, Tunis, Tunisia

**Keywords:** salivary gland, liver metastasis, adenoid cystic carcinoma

## Abstract

Adenoid cystic carcinoma is an uncommon malignant neoplasm of the salivary gland. Liver metastasis from salivary gland cancer is a rare situation. In this article, we report the case of a 29-year-old woman treated 5 years previously for adenoid cystic carcinoma of the parotid gland by surgery and radiotherapy, who presented for a large hypervascularized hepatic metastasis of 20 cm. After 3-cycle chemotherapy stability, hepatic surgery was successfully performed. The patient maintained disease-free period of 12 months after the surgical treatment. This rare case represents a therapeutic challenge for oncologists and surgeons. Through this case and a review of the literature, we try to better detail the management of this uncommon entity.

## Introduction

Adenoid cystic carcinoma (ACC) is a rare malignant neoplasm of the salivary gland.^1^

The main characteristics of ACCs are the slow growth, multiple local recurrences, and a high frequency of distant metastases.^2^ The common pattern of disease spread is hematogenous, and often the sites of metastasis are the bone, viscera, lung,^1,3^ and, only rarely, liver.^2^

We present a case of solitary liver metastases occurring 5 years after surgery for ACC of the parotid gland. Furthermore, we review the literature concerning the management of liver metastases from ACC of the parotid gland.

## Case Presentation

A 29-year-old woman was treated in 2013 for ACC of the parotid (T3N0M0). She had parotidectomy of the left parotid gland with ipsilateral selective lymph node dissection level II to IV preserving the facial nerve. The treatment was followed by external beam irradiation to the parotid area and the neck. Afterward, she was regularly followed-up in our consultation. Five years after primary surgery, the patient consulted with pain in the right hypochondrium. In the clinical examination, the patient was anicteric, and abdominal examination found an irregular mobile considerable tumefaction of the left liver. There was no recurrence in the surgical site and neck.

**Figure 1.**
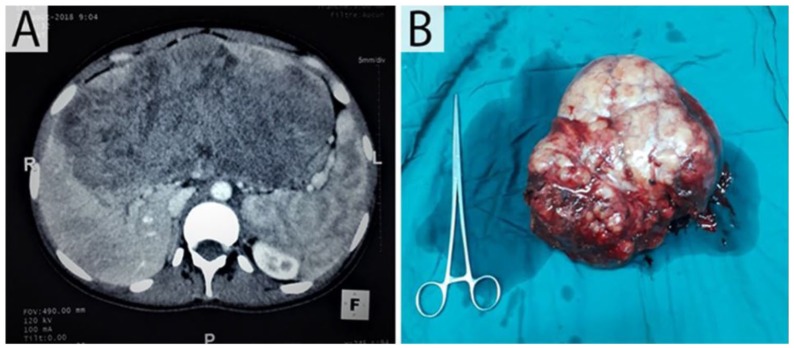
(A) Contrast-enhanced computed tomography images showed a solitary focal large lesion measuring 21 × 09 cm with contrast agent enhancement of the left lobe of the liver. (B) Specimen of wedge resected liver: voluminous exophytic tumor; white grayish color measuring 21 × 19 × 12 cm.

A computed tomography (CT) scan revealed a single liver lesion that was suspicious of metastasis (Figure 1A). The CT scan of the brain, head, neck, and chest did not reveal other distant metastasis.

Magnetic resonance imaging of the liver affirmed a solitary liver lesion. It was a heterogeneous, large mass measuring 21 × 9 cm with a contrast agent enhancement of the left lobe of the liver that was suspicious of metastasis. A CT-guided needle biopsy of the mass was done and revealed a metastatic ACC of the parotid. A multidisciplinary staff decision was made to perform chemotherapy to down-size the lesions, followed by hepatic metastasectomy. The young age and the presence of a single metastatic localization were the arguments justifying the hepatic surgery on our patient.

After 3 cycles of Navelbine-CDDP-based chemotherapy and in view of the stability of the lesion objectified on the CT scan, we decided to do a chemo-embolization, which failed due to extensive vascularization and of the technical difficulties.

After an evaluation of liver function, calculated at 40% by volumetric CT, the patient had a laparotomy and we found a voluminous exophytic tumor. It was white grayish color measuring 21 × 19 × 12 cm. The mass was appended to the liver segment (III, IV) surface. Wedge resection of the tumor while keeping sufficient liver parenchyma was done. Intraoperative ultrasound was performed and revealed no more lesions.

The patient had no postoperative complications. There was no liver dysfunction, and she left the hospital on the 15th postoperative day in good health condition.

**Figure 2.**
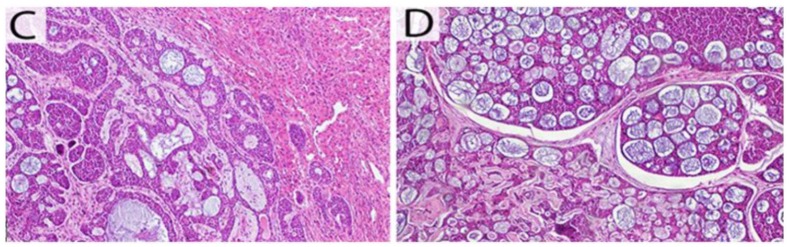
(C) The microscopic appearance showed that the tumor mass is pushing out toward the hepatic tissue (hematoxylin and eosin [H&E]). (D) The tumor was organized in cribriform growth pattern displaying several prominent pseudocysts surrounded by basaloid cells with hyperchromatic-angulated nuclei (H&E).

A histologic examination showed that the tumor measured 21 × 19 × 12 cm with disease-free surgical margins. The microscopic appearance showed that the tumor mass is pushing out toward the hepatic tissue. The tumor was organized in cribriform growth pattern displaying several prominent pseudocysts surrounded by basaloid cells with hyperchromatic-angulated nuclei. These pathological findings are suggestive of metastases from the ACC (Figure 2C and D).

At the 6-month postoperative follow-up examination, magnetic resonance imaging of the abdomen showed that the patient is currently disease-free. One year after the surgery, she has regularly been followed-up at the consultation and has no signs of recurrence.

## Discussion

Usually ACC has a slow growth. It is characterized by the frequency of local recurrences and a high rate of distant metastases.^2^ Distant metastases most frequently were localized in the lung, followed by bone, liver, skin, breast, and, rarely, the brain.^3-5^

The occurrence of liver metastases is often synchronous or metachronous with metastasis to other organs like the lung.^1^ Generally, metastases from ACC occurred many years after the diagnosis of the primary tumor^1,2,6^ and could persist asymptomatic for an extended period.^5^

According to our current knowledge, only 3 cases of isolated liver metastasis that presented as the first clinical sign from parotid ACC were reported.^7-9^

Few studies of isolated metastatic liver were reported in the literature. Their management was still debatable. The role of surgery was not clear and some study showed no benefit on overall survival.^2,6,10^

Often, it is hard to show whether liver resection could provide a survival benefit because of the frequent association of liver metastases with other metastatic sites.

Our patient was young, and she had developed a unique metastasis in her liver 5 years after the diagnosis of ACC of the parotid. Because of the large size of this tumor, chemotherapy was tried initially. However, it did not have any effect. Surgery was the only possible treatment, despite the high risk to which she was exposed.

We found in the literature 2 cases^2,10^ that were similar to ours. Both cases had isolated liver metastases and showed resistance to chemotherapy as first-line therapy.

In the case first reported by Balducci et al,^2^ the patient had multiple liver metastases with a low response to chemotherapy, so a right extended hepatectomy after a portal vein embolization was done. The patient was disease-free for 18 months before the final recurrence.

Karatzas et al^10^ described the second case of liver metastases, as the initial sign of an ACC of the left submandibular gland. She had multifocal liver lesions and showed resistant to systemic chemotherapy. She received a combination of a doxorubicin-eluting bead chemoembolization, intraoperative and percutaneous radiofrequency ablation, and radiofrequency-assisted surgical resection. At 1-year follow-up, the patient had no recurrence.

Scuderi et al^11^ reported one case of recurrent liver metastasis that was efficiently achieved by a laparoscopic approach. The patient remained disease-free for 1 year after the surgical resection.

Unfortunately, considering the rare cases of isolated liver metastases, it is difficult to conclude whether liver metastasectomy could be advantageous. Some studies had proposed no further treatment (radiotherapy or chemotherapy) when asymptomatic distant metastases were detected.^4,5,12^ Due to the slow growth rate of ACC, the role of chemotherapy, in a metastatic situation, is still inefficient.^4,13,14^ However, surgical excision, with adequate margins, remains the only possible therapeutic option.^5^

## Conclusion

To conclude, liver metastasis from salivary gland cancer is an uncommon situation. Due to the absence of consensus concerning the appropriate treatment, a multidisciplinary approach was necessary to manage this rare and aggressive neoplasm.

Surgical resection of the metastasis is recommended particularly when metastasis is solitary in order to improve survival.
